# Perturbation of specific pro-mineralizing signalling pathways in human and murine pseudoxanthoma elasticum

**DOI:** 10.1186/1750-1172-9-66

**Published:** 2014-04-29

**Authors:** Mohammad J Hosen, Paul J Coucke, Olivier Le Saux, Anne De Paepe, Olivier M Vanakker

**Affiliations:** 1 Center for Medical Genetics, Ghent University Hospital, Ghent, Belgium; 2 Department of Genetic Engineering and Biotechnology, Shahjalal University of Science and Technology, Sylhet 3114, Bangladesh; 3 Department of Cell and Molecular Biology, John A. Burns School of Medicine, Honolulu, HI, USA

**Keywords:** Pseudoxanthoma elasticum, Ectopic mineralization, Elastic fibres, Osteogenic signalling pathway, BMP2-SMADs-RUNX2, TGFβ signalling, Canonical Wnt pathway, Apoptosis, Endoplasmic reticulum stress

## Abstract

**Background:**

Pseudoxanthoma elasticum (PXE) is characterized by skin (papular lesions), ocular (subretinal neovascularisation) and cardiovascular manifestations (peripheral artery disease), due to mineralization and fragmentation of elastic fibres in the extracellular matrix (ECM). Caused by mutations in the ABCC6 gene, the mechanisms underlying this disease remain unknown. The knowledge on the molecular background of soft tissue mineralization largely comes from insights in vascular calcification, with involvement of the osteoinductive Transforming Growth Factor beta (TGFβ) family (TGFβ1-3 and Bone Morphogenetic Proteins [BMP]), together with ectonucleotides (ENPP1), Wnt signalling and a variety of local and systemic calcification inhibitors. In this study, we have investigated the relevance of the signalling pathways described in vascular soft tissue mineralization in the PXE knock-out mouse model and in PXE patients.

**Methods:**

The role of the pro-osteogenic pathways BMP2-SMADs-RUNX2, TGFβ-SMAD2/3 and Wnt-MSX2, apoptosis and ER stress was evaluated using immunohistochemistry, mRNA expression profiling and immune-co-staining in dermal tissues and fibroblast cultures of PXE patients and the eyes and whiskers of the PXE knock-out mouse. Apoptosis was further evaluated by TUNEL staining and siRNA mediated gene knockdown. ALPL activity in PXE fibroblasts was studied using ALPL stains.

**Results:**

We demonstrate the upregulation of the BMP2-SMADs-RUNX2 and TGFβ-2-SMAD2/3 pathway, co-localizing with the mineralization sites, and the involvement of MSX2-canonical Wnt signalling. Further, we show that apoptosis is also involved in PXE with activation of Caspases and BCL-2. In contrast to vascular calcification, neither the other BMPs and TGFβs nor endoplasmic reticulum stress pathways seem to be perturbed in PXE.

**Conclusions:**

Our study shows that we cannot simply extrapolate knowledge on cell signalling in vascular soft tissue calcification to a multisystem ectopic mineralisation disease as PXE. Contrary, we demonstrate a specific set of perturbed signalling pathways in PXE patients and the knock-out mouse model. Based on our findings and previously reported data, we propose a preliminary cell model of ECM calcification in PXE.

## Background

**Figure 1:**
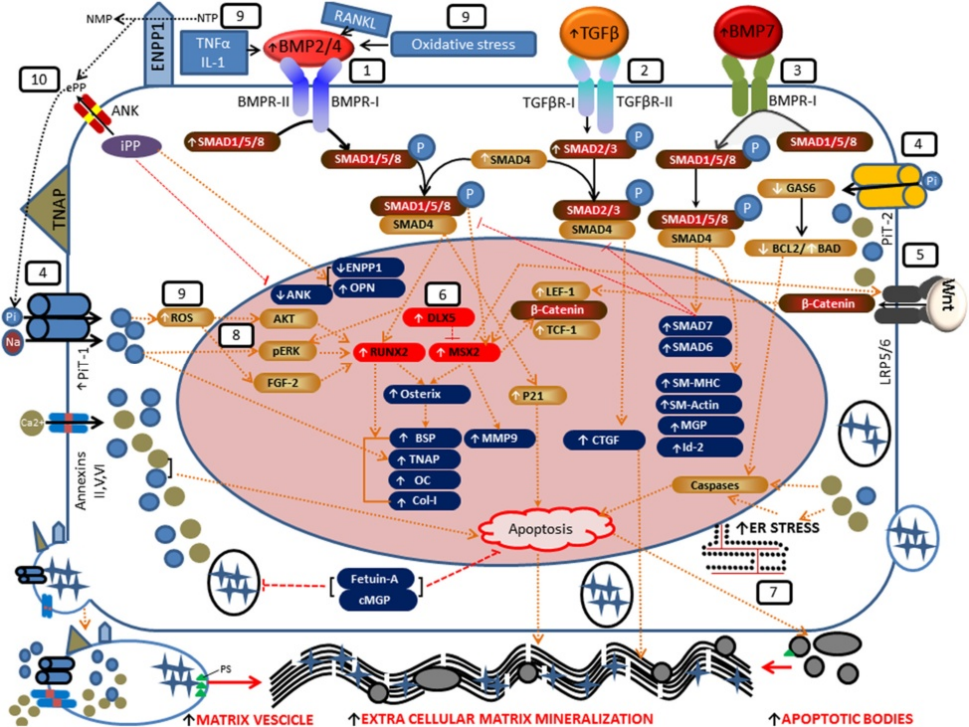
**Schematic representation of the pathways (1–10) involved in vascular soft tissue mineralization.** Vascular calcification is the result of a transdifferentiation process of vascular smooth muscle cells (VSMCs) to chondrocyte-like or osteoblast-like cells [18]. (1) BMP2 and BMP4 induce osteogenic differentiation through SMAD dependent signal transduction. BMP2/4 ligands bind with BMP receptors BMPR-I and BMPR-II; BMPR-II then phosphorylates and activates BMPR-I. Phosphorylated BMPR-I subsequently phosphorylates SMAD1, SMAD5 and SMAD8 (SMAD1, 5, 8) which associate with SMAD4 to form the heteromeric pSMAD1,4,5,8. This facilitates nucleation, where they regulate the pro-osteogenic runt-related transcription factor RUNX2 [19]. Downstream targets of RUNX2 include osterix (OSX), alkaline phosphatase (ALPL or TNAP), osteopontin (OPN), osteocalcin (OC) and collagen type-1 (Col-1) [20]. (2) TGF-βs (1-2-3) bind to TGF-β receptor type I or II, resulting in phosphorylation of SMAD2/3, coupling with SMAD4 and translocation into the nucleus [21-23], where they trigger targeted gene expression of connective tissue growth factor (CTGF) [24-26]. (3) BMP7 activates endogenous SMAD1,5,8 and forms a SMAD1,4,5,8 complex, facilitating nucleation and stimulation of VSMC specific genes (SM-MHC, SM actin, MGP, Id-2). Through SMAD6 and SMAD7, BMP7 also inhibits BMP2 and TGF-β signalling [27]. (4) Elevated inorganic phosphate (Pi) enters the cell via sodium-dependent phosphate co-transporters PiT-1 and 2, resulting in downregulation of mineralization inhibitors, apoptosis (through downregulation of GAS6 and BCL-2, thus activating caspases), and upregulation of RUNX2 through ERK1/2 activation [28-30]. (5) MSX2 upregulates different Wnt receptors, causing stabilization of β-catenin, which after nucleation increases TCF-1/LEF-1 dependent gene expression. This in turn can upregulate MSX2 expression [31]. (6) DLX5 drives RUNX2 expression and osteogenic differentiation, and can abrogate osterix induction by BMP2. DLX5 also negatively regulates the activity of MSX2 [32]. (7) ER stress initiates a cascade of chaperone proteins leading to activation of caspases such as Caspase 12 and apoptosis (so-called unfolded protein response) [33]. (8) BMP2 can directly increase expression of ERK1/2, thus increasing RUNX2 expression and SMC phenotypic transition toward osteochondro-progenitors [34]. (9) Oxidative stress and inflammatory mediators respond with higher BMP2/4 secretion in VSMCs calcification process [35,36]. (10) Tissue nonspecific alkaline phosphatase (TNAP or ALPL) has a major role both as a PPiase and as an ATPase/ADPase and thus participates in the calcification process by restricting the concentration of extracellular PPi, a calcification inhibitor. Nucleotide pyrophosphatase phosphodiesterase 1 (NPP1) works as a backup for PPiase and ATPase, especially in the absence of [27].

Pseudoxanthoma elasticum (PXE; OMIM # 264800) is an autosomal recessive systemic connective tissue disease affecting the extracellular matrix (ECM) of multiple organs [1]. It is characterized by dermal (papular lesions in flexural areas), ocular (angioid streaks, subretinal neovascularisation and haemorrhage) and vascular symptoms (coronary and peripheral vascular disease) which result from mineralization and fragmentation of elastic fibres. PXE is caused by mutations in the ABCC6 (ATP-binding cassette subfamily C member 6) gene, encoding a transmembrane ATP driven organic anion transporter, the substrate of which is currently unknown. The biological mechanisms of ectopic mineralization in PXE, including the exact relationship with the defective ABCC6 transporter remain unclear [2]. Current knowledge on the molecular background of soft tissue mineralization largely comes from insights in vascular calcification (Figure 1). Murine models of calcified vasculopathies demonstrate that signalling pathways involved are those required for the physiological development of bone and cartilage, influencing gene transcription, apoptosis, matrix vesicle formation, endoplasmic and oxidative stress. Main protagonists are the osteoinductive Transforming Growth Factor beta (TGFβ) family (TGFβ1-3 and Bone Morphogenetic Proteins [BMP]), together with ectonucleotides (ENPP1), Wnt signalling and a variety of local and systemic calcification inhibitors, many of which have been previously associated with PXE, such as matrix Gla protein (MGP), osteocalcin (OC), bone sialoprotein (BSP or osteopontin), osteoprotegerin (OPG) and fetuin-A [3-9]. MGP is a protein belonging to the family of so-called “gla-proteins”, because of the presence of “gla-residues” which need to undergo gamma-carboxylation for activation of the protein. This carboxylation process is performed by the GGCX (gamma-glutamylcarboxylase) enzyme in the so-called ‘vitamin K (VK)-cycle’, as VK is an essential co-factor for this post-translational modification [10]. MGP serves as mineralization inhibitor via direct repression of bone morphogenetic protein-2 (BMP2), an osteo-inductive member of the TGF-β family of growth factors [11-13], which has also been implicated in directing soft tissue calcification [14]. We and others have previously shown that MGP is abundantly present in calcified PXE tissues in its uncarboxylated or inactive form and that the loss of MGP repression on BMP2 results in an upregulation of BMP2 in the middermis of PXE patients [5]. The observation of low VK1 serum levels in PXE patients was suggested as a contributing factor leading to this inefficient carboxylation of MGP. Besides local inhibitors, PXE patients were shown to have a deficiency of the systemic mineralization antagonist Fetuin-A [15]. Recently, the role of the ectonucleotide pyrophosphatase/phosphodiesterase 1 or ENPP1 was confirmed as mutations in the encoding gene can also result in PXE [16,17].

However, limited information is available on the key cellular pathways of soft tissue mineralization, the TGFβ superfamily-related signalling - its ligands (TGFβ1-3, BMPs), its receptors (TGFβR1/2, BMPRI/II) and its intracellular signal transducers (SMADs - Mothers Against Decapentaplegic homolog proteins) -, Wnt signalling, apoptosis or ER stress, in PXE. In the heart of Abcc6 −/−mice, the BMP responsive transcription factors Smad1/5/8 were found to be upregulated, implying deregulation of BMP signalling pathways. Though no other tissues were examined, the authors concluded that further study of the BMP signalling axis may be of importance in the study of PXE [37]. The potential importance of these signalling pathways was recently endorsed by Sowa et al. demonstrating ectopic expression of RUNX2 in calcified cardiac tissue of the Dyscalc1 mice, in which an Abcc6 splice variant results in Abcc6 transporter deficiency [38]. These findings suggest that signalling pathways implicated in calcified vasculopathies may be relevant for PXE, though it may seem presumptuous to assume that their involvement is identical in a complex multisystem calcification disease such as PXE. In this study, we have investigated the relevance of the signalling pathways described in vascular soft tissue mineralization in the PXE knock-out mouse model and in PXE patients. We demonstrate perturbance of several but not all effectors of vascular mineralization. We have combined our results and literature data on PXE to propose a preliminary cell-based model of ECM mineralization in PXE as a starting point for cellular research in this complex disease.

## Methods

### Ethics statement

This study was approved by the Ethical Committee of the Ghent University Hospital. Informed consent was obtained from all patients and the Declaration of Helsinki protocols were followed.

### Abcc6 KO mice

The mouse model for PXE has been developed by targeted ablation of the Abcc6 gene [39]. The mice were maintained in the Animal facility of the Department of Cell and Molecular Biology, John A. Burns School of Medicine, University of Hawai’i. These knockout mice recapitulate the histopathological and ultrastructural features of human PXE. In addition, a prominent mineralization of the connective tissue capsule surrounding the hair follicles in vibrissae of this mouse model has been observed. This feature has been proposed as an early biomarker of the overall mineralization process in PXE [9]. The IHC experiments in this study were performed on paraffin embedded tissues of the eyes (Bruch’s membrane) and whiskers of the Abcc6 −/−mice.

### PXE patients

Dermal tissues and fibroblast cultures were obtained through full thickness skin biopsies in macroscopic skin lesions from clinically and molecularly confirmed PXE patients followed in the PXE Clinic of the Ghent Center for Medical Genetics.

Fibroblasts of 8 PXE patients and 5 healthy age- and sex-matched controls were cultured in Dulbecco’s modified Eagle’s medium (DMEM) containing 10% Fetal Calf Serum, 1% penicillin/Streptomycin, 1% Kanamycin, 1% non-essential amino acid and 0.1% fungizone. Cultures were maintained by refreshing media twice a week and incubated at 37°C (5% CO_2_). First, fibroblasts were grown in T25 cm^2^ flask; when 100% confluent, fibroblasts were spliced and transferred in T75 cm^2^ flask. When 100% confluent, cells were spliced and grown in 60 mm petri-dishes. Fully confluent 60 mm petri-dishes were scraped and centrifuged to extract the tissues and stored at −80°C.

### Histological analysis

Alizarin red staining (ARS): Five μm sections from muzzle skin containing whiskers (or vibrissae) from Abcc6−/−mice and lesional skin sections from PXE patients were stained with 2% (pH 4.2, freshly prepared) ARS (Sigma-Aldrich, Belgium). To observe ARS in fibroblast cultures (n = 3), cells were fixed in 10% phosphate-buffered formalin, subsequently washed with PBS (pH 7.4) and stained with Alizarin Red solution (40 mM Alizarin Red-Tris–HCl, pH 4.1) at room temperature (RT) for 10 min. After washing three times with PBS, cells were mounted with vectashield (Vectastain kit, Labconsult, CA).

Alkaline phosphatase (ALPL) staining: To detect ALPL activity, control (n = 3) and PXE (n = 3) fibroblasts were cultured in an 8 well chamber slide, fixed with 0.4% cold paraformaldehyde (10 min.), rinsed with ALPL solution (100 mM Tris–HCl, pH 9.5, 100 mM NaCl, 10 mM MgCl_2_) and stained with a 1% BM Purple solution (Roche Molecular Biochemicals, Belgium) at 37°C (30 min.). After light microscopical evaluation (Zeiss, Germany), images were taken for all experiments using Axiovision Reflected Light 4.6 software (Carl Zeiss MicroImaging, GmbH, Germany).

### Immunohistological analysis

Immunohistochemistry (IHC) was performed on formalin-fixed and paraffin embedded whisker and eye tissues (5 μm) of Abcc6 KO mice and human dermal tissues prepared from lesional skin biopsies, using primary antibodies against BMP2 (Abcam, USA), pSMAD1 (Abcam, USA), pSMAD2 (Cell Signalling Technology, The Netherlands), pSMAD4, 5 (Abcam, USA) and pSMAD8 (Santa Cruz Biotechnology, Inc., Germany), pSMAD1-5-8 (an antibody recognizing SMAD1 only when dually phosphorylated at Ser463 and Ser465, as well as phosphorylated SMAD5 and SMAD8; Abcam, USA), RUNX2 (M70, Santa Cruz Biotechnology, Inc., Germany), CTGF (Abcam, UK), Caspase 3 (BIOKE, Cell Signalling Technology, The Netherlands) and pERK1/2 (Abcam, UK). Labelling was always performed on slides adjacent to those with proven mineralization on Alizarin Red staining (Sigma-Aldrich, Belgium). Each labelling was done on 5 (patients, mouse tissues) or 3 (human controls) slides. Briefly, antigens were unmasked after deparaffinization, using 1 mM EDTA (pH 8, boiled for 20 min.), cooled down (30 min. at RT) and subsequently antibody binding steps were performed by washing sections in distilled water (3 × 5 min.), 3% hydrogen peroxide (1 × 10 min.), distilled water (2 × 5 min.) and in TBS-T (1 × 5 min.). Sections were blocked with 5% BSA (in TBST) for 1 h. at RT. After removal, sections were incubated with primary antibodies (1:100 dilution in TBST + 5% Bovine Serum Albumin) overnight at 4°C in a moist chamber. After washing, the secondary antibody (Rabbit IgG) was added according to manufacturer’s recommendation (Vectastain kit, Labconsult, CA). After washing, tissue sections were incubated with ABC reagent (45 min. at RT; Vectastain kit, Labconsult, CA) and subsequently treated with AEC reagent (30 min.) or DAB (Vectastain kit, Labconsult, CA). Slides were washed mounted with cover slips. Tissue sections were evaluated using light microscopy (Zeiss, Germany). Images were taken using Axiovision Reflected Light 4.6 software (Carl Zeiss Microlmaging, GmbH, Germany).

### Fluorescent immunohistochemistry

To detect co-localization of RUNX2-Caspase 8, fluorescent immunohistochemistry was performed on PXE skin tissue (n = 5). Tissue sections were deparaffinized, and blocked with 5% BSA (1 h), incubated with an anti-rabbit polyclonal RUNX2 antibody (1:200; M 70, Santa Cruz Biotechnology Inc., Germany) and an anti-mouse monoclonal Caspase 8 antibody (1:200; BIOKE, Cell Signalling Technology, The Netherlands) for 2 hrs. After removal of the primary antibody, the sections were incubated with the secondary antibody Cy3 (anti-rabbit, 1:100; GE Healthcare, Germany) and Alupa 488 (anti mouse, 1:100; Life Technologies Europe) for 1 h.

All tissues were mounted with vectashield with DAPI (Vectastain kit, Labconsult, CA) and images were taken under the fluorescent microscope (Axioplan 2 Imaging, Zeiss, Germany).

### Gene expression quantification by qPCR

qPCR analysis was performed for key genes involved in the BMP2/BMP4-SMADs-RUNX2 signalling pathway (RUNX2, BMP2, BMP4, SMAD1, SMAD4, SMAD5, SMAD8), the downstream RUNX2 target gene Osterix and ALPL, genes in the TGFβ signalling pathway (TGFβ-1, TGFβ-2, TGFβ-3, SMAD2, SMAD3 and CTGF), genes of the MSX2-Wnt pathway (MSX2, DLX5, LEF-1, TCF-1 and β-catenin), the inorganic phosphate transporter PiT-1, genes involved in apoptosis pathways (P21, GAS6, BCL-2, Caspase3), and genes for endoplasmic reticulum stress (CHOP, BIP, XBP1, IRE1, ATF4, ATF6, GAD34, JNK, XBP-S). RNA was isolated from fully confluent fibroblasts using the RNeasy® kit (Qiagen, GmbH, Germany) according to manufacturer’s recommendation. To purify the RNA from any DNA that may be present, it was incubated with DNase (15 min. at RT). Concentration of total RNA was measured via the DropSense-96 multichannel spectrophotometer (Micronic North America, USA). cDNA was prepared from 2 μg of RNA using the iScript cDNA synthesis kit (Bio-Rad Laboratories, CA) and diluted 10-fold. qPCR was performed on control (n = 5) and PXE fibroblasts (n = 8) using HPRT1 (hypoxanthine phosphoribosyl transferase 1) and YWHAZ (tyrosine 3-monooxygenase/tryptophan 5-monooxygenase activation protein, zeta isoform) as reference genes and the FastStart Universal Probe Maser Mix (Roche Applied Science, GmbH, Germany) in the Roche-LightCycler®480 real-time PCR system (Roche Applied Science, Belgium). qPCR primers are listed in Additional file 1: Table S1. Real time PCR data were analysed via the qbasePLUS software (Biogazelle, Belgium).

### In situ cell death detection

In vitro evaluation of fibroblast cell death was performed via the in situ cell death detection (TUNEL - Tdt-mediated dUTP Nick-End Labelling) kit (Roche Diagnostics, GmbH, Germany). PXE fibroblasts were grown in 75 cm^2^ flask until 100% confluent. Concentration of the cells/ml was determined using an automated cell counting machine (Cellometer®auto T4, Nexcelom Bioscience, USA). Forty thousand cells per well were distributed on an 8 well plate. After 2 days, cells were washed with PBS (pH 7.4). Cells were stained with the TUNEL kit according to the manufacturer’s protocol after 24, 72 and 120 hrs. respectively. Percentage of cell death was counted under fluorescent microscope (Axioplan 2 imaging, Zeiss, Germany). For each cell culture (n = 6 for patients and 5 for controls), 10 microscopic fields were evaluated.

### RUNX2 siRNA transfection

To evaluate the expression of RUNX2 at different time points (24, 48 and 72 hrs.) after siRNA transfection, 2 × 10^5^ PXE fibroblasts (n = 6 cultures) were seeded in 6 well plates. After 24 hrs. cells were transfected with 6 μl (2 μM) of RUNX2 siRNA (sc-37145, sc-36868, sc-29528, Santa Cruz Biotechnology, Inc, Europe) or scrambled RNA (sc-37007, Santa Cruz Biotechnology, Inc, Europe) according to the manufacturer’s recommendations. Expression of RUNX2 after siRNA transfection was evaluated by qPCR (using primer sc-37145-PR, Santa Cruz Biotechnology, Inc, Europe) after 24, 48 and 72 hrs. (n = 2 cultures) TUNEL assay was performed as described above, respectively at 24, 48 and 72 hrs. after siRNA transfection (n = 6 cultures).

### Statistical analysis

All data were analysed using the statistical software S-PLUS 8 (Insightful, Washington). Normality distribution of the data was evaluated by the Kolmogorov-Smirnov test. Differences between groups were compared using the one-sample *t*-test. Significance was considered at a confidence level of 0.95 and p < 0.05.

## Results

### The BMP2-SMADs-RUNX2 pathway is upregulated in human and murine PXE

**Figure 2:**
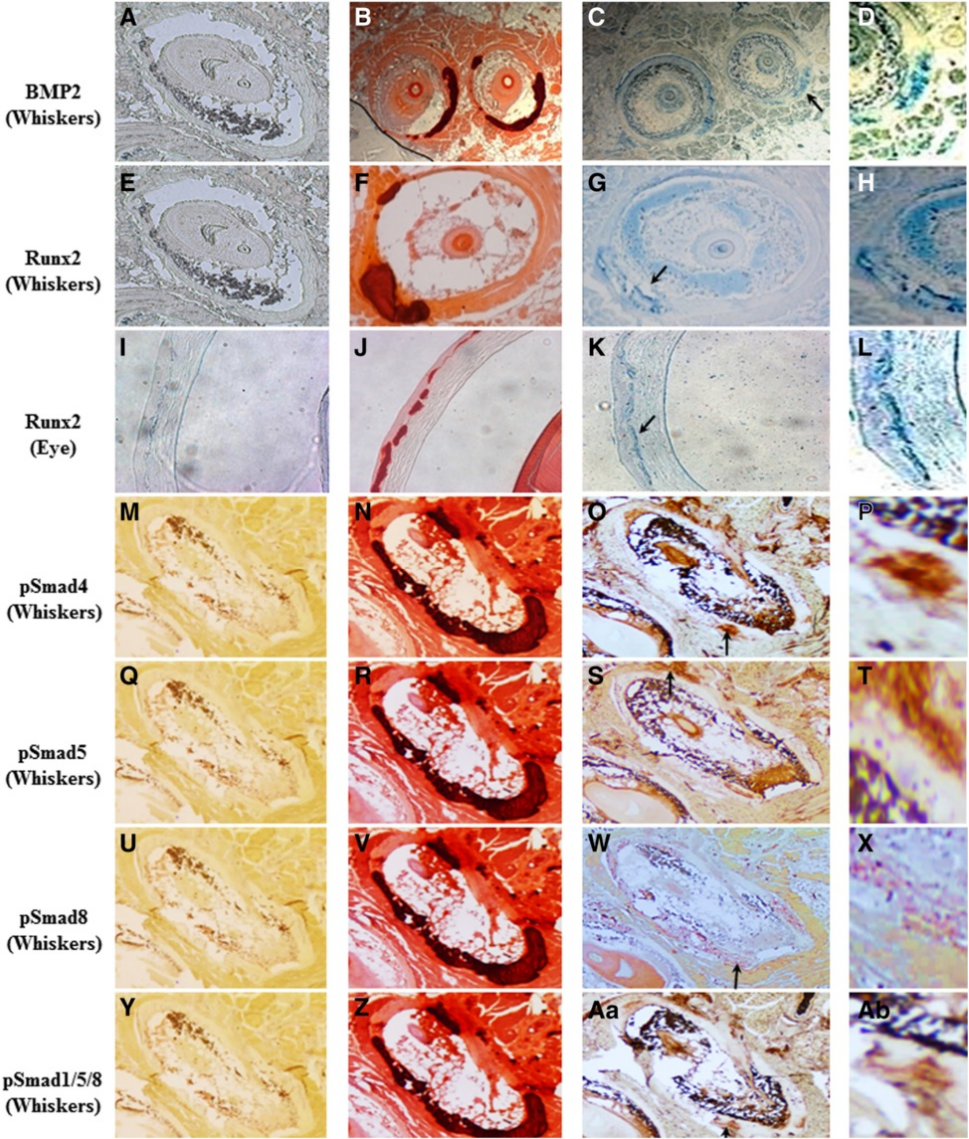
**Staining results for the BMP2-SMADs-RUNX2 pathway in whisker and eye sections from the Abcc6 KO mouse (×10).** Negative controls are shown in the first column **(A, E, I, M, Q, U, Y)**. Alizarin Red stainings confirms profound calcification in the connective tissue capsule of the whiskers **(B, F, N, R, V** and **Z)** and Bruch’s membrane of the eye **(J)**. Immunohistochemical staining of adjacent slides with antibodies against respectively Bmp2 **(C)**, Runx2 **(G, K)**, pSmad4 **(O)**, pSmad5 **(S)**, pSmad8 **(W)** and pSmad1/5/8 (Aa), shows positive staining for all, co-localizing with the mineralization foci. Arrowed areas of positive staining have been magnified (×20). Scale bar = 100 μm. n = 5 each.

**Figure 3:**
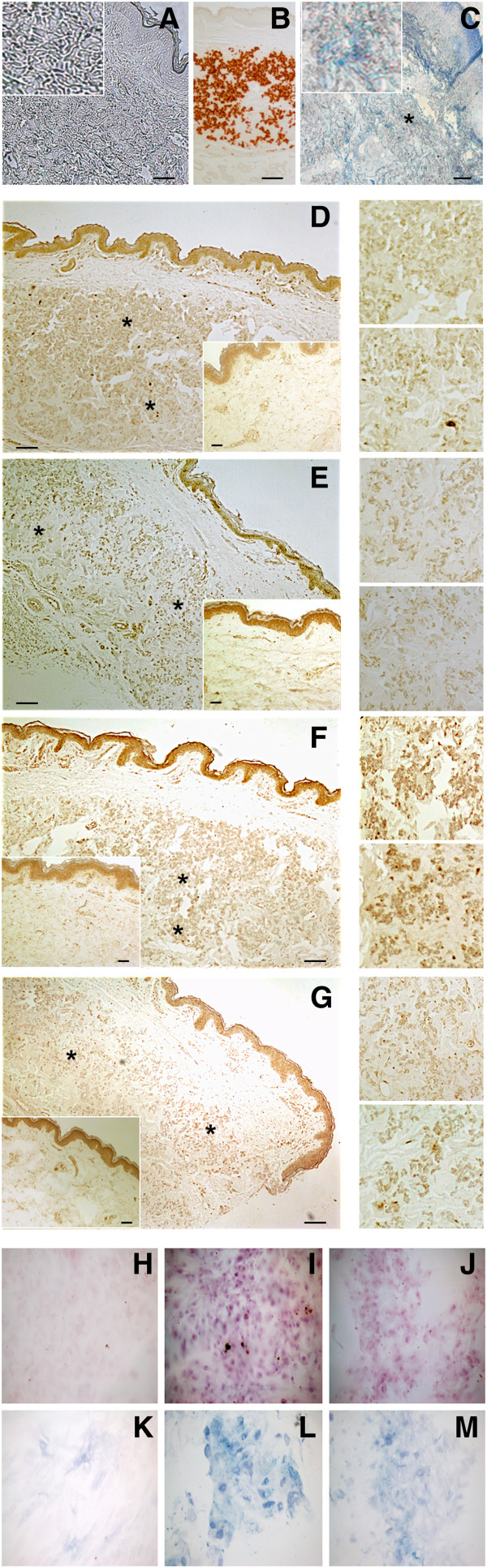
**Immunohistochemical labelling results of the SMAD-RUNX2 pathway in human PXE skin (n = 5 each) and fibroblasts (n = 8 each) compared to controls (n = 3 each) (×10).** The top-left panel shows absence of staining in controls **(A)**, positive middermal staining for Alizarin red **(B)** and RUNX2 **(C)** in PXE skin. The top-right and middle two panels (**D** to **H**) depicts staining for pSmad4 **(D)**, pSmad5 **(E)**, pSmad8 **(F)** and pSMAD1/5/8 **(G)** with positive labelling in the middermis of PXE patients compare to no labelling in the negative controls (inserts). The lower panel reveals positive labelling for Alizarin Red **(J, K)** and ALPL **(L, M)** in PXE fibroblast cultures (n = 3) compared to controls (n = 3) **(I, L)**. Areas marked with asterisk are enlarged (×40). Scale bar = 100 μm.

One of the most important pathways in vascular calcification involves BMP2-SMADs-RUNX2 signalling, as RUNX2 is considered a master regulator of mineralization. To study the relevance of this pathway in PXE, first IHC staining of key proteins of this pathway was performed on the vibrissae and eyes of the Abcc6 KO mouse. Labelling for BMP2, pSMAD1, pSMAD4, pSMAD5, pSMAD8, pSMAD1-5-8 and RUNX2 revealed increased expression in the connective tissue capsule around the whiskers and in Bruch’s membrane of the eye compared to the wild type. These labellings co-localized with mineralization foci as seen on Alizarin Red stains (Figure 2). Subsequently, identical IHC stains were performed on human PXE dermis, which revealed identical results for all proteins. Positive staining in the human samples was confined to the mid-dermal skin area of elastic fibre mineralization compared to no staining in controls (Figure 3, A-G).

**Figure 4:**
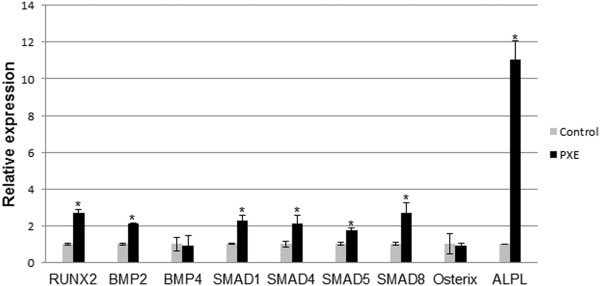
**Relative expression of components of BMP2-SMADs-RUNX2 signalling including RUNX2, BMP2, BMP4, SMAD1, SMAD4, SMAD5, SMAD8, Osterix, and Alkaline Phosphatase (ALPL).** Relative expression is shown in human PXE fibroblasts and control cells. A significant upregulation of all genes except BMP4 and Osterix was observed compared to controls. (n = 8 and 5 for patients and controls respectively). *p < 0.05.

qPCR experiments performed for RUNX2, BMP2, BMP4, SMAD1, SMAD4, SMAD5, SMAD8, Osterix and ALPL confirmed a significant upregulation of RUNX2, BMP2, SMAD1, SMAD4, SMAD5, SMAD8 and ALPL in PXE fibroblasts compare to healthy controls (Figure 4), whereas expression of BMP4 and Osterix remained same as control. ALPL, one of the target genes of RUNX2, encodes the enzyme alkaline phosphatase (ALPL) which is an inducer of mineralization and showed the highest upregulation compared to controls (more than 3-fold; p < 0.05), whereas RUNX2 showed a more than 2-fold increase in expression (p < 0.05). The ALPL upregulation was also demonstrated on fibroblast cultures of PXE patients, where staining for Alizarin Red and ALPL showed positive labelling for both in PXE cells compared to controls (Figure 3, H-M).

### MSX2 expression is influenced by increased BMP2 and activates LEF-1/TCF-1 transcription

**Figure 5:**
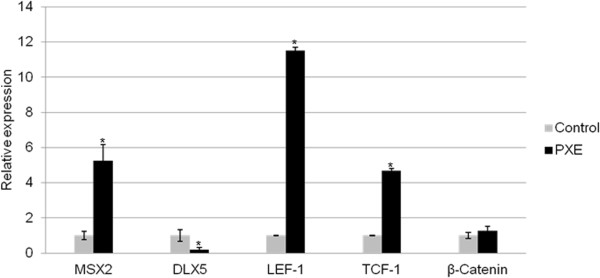
**Relative expression of mediators in MSX2-Wnt signalling (MSX2, DLX5, LEF-1, TCF-1 and β-catenin).** qPCR reveals a marked upregulation of MSX2, LEF-1 and TCF-1 as well as significant downregulation of DLX5 in human PXE fibroblasts compared to control cells. β-catenin is not differentially expressed in PXE fibroblasts. (n = 8 and 5 for patients and controls respectively). *p < 0.05.

Besides SMADs-RUNX2 signalling, BMP2 can also upregulate MSX2. The expression of MSX2 was indeed significantly upregulated in PXE fibroblast compared to health controls (Figure 5). This was further corroborated by a decreased DLX5 expression, a known repressor of MSX2. In vascular calcification, MSX2 overexpression stimulates nuclear localisation of β-catenin, thus activating transcription of LEF-1 and TCF-1. Indeed, qPCR experiments reveal that expression of LEF-1 and TCF-1 was upregulated in PXE fibroblasts compared to controls, while β-catenin was expressed within normal limits (Figure 5).

### Human and murine PXE manifest increased TGFβ-2 signalling

**Figure 6:**
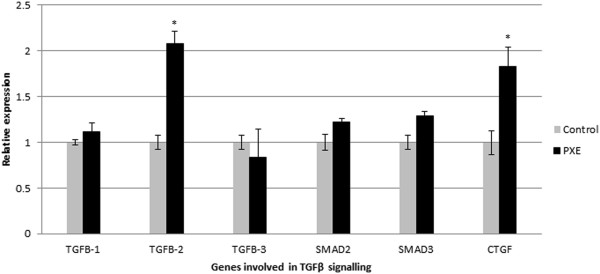
**qPCR result for markers involved in TGFβ signalling pathway (TGFβ-1, TGFβ-2, TGFβ-3, SMAD2, SMAD3 and CTGF).** While relative expression of TGFβ-1 and −3 are normal, upregulation of TGFβ-2 and CTGF can be seen in human PXE fibroblasts compared to control cells. (n = 8 and 5 for patients and controls respectively). *p < 0.05.

**Figure 7:**
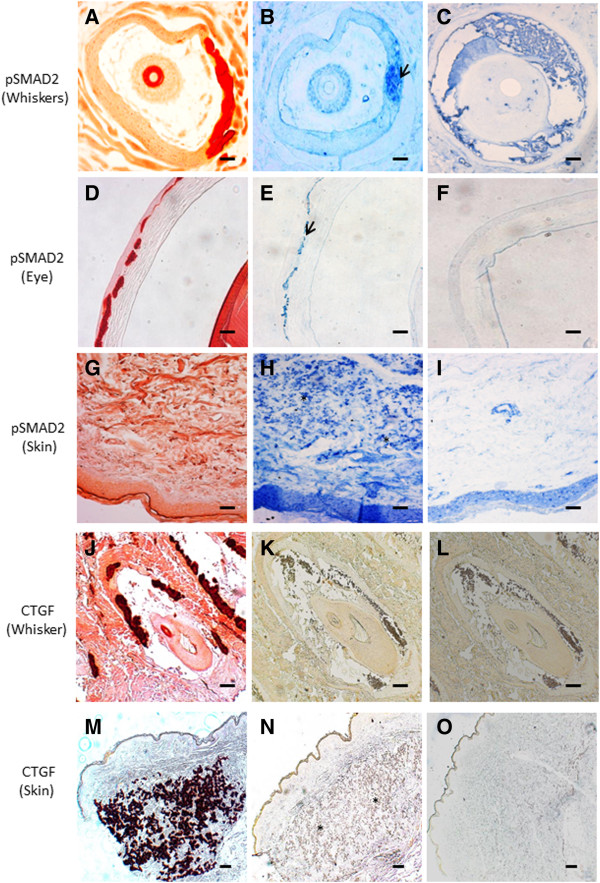
**Immunohistochemical labelling of key mediators of TGFβ-2 signalling in whisker and eye sections from the abcc6 knockout mouse (and skin tissues from PXE patients (×10).** Alizarin Red staining visualizes calcification in the whiskers **(A, J)**, Bruch’s membrane of the eye **(D)** and mid-dermis of the skin tissues **(G, M)**. Immunohistochemical staining of adjacent slides with antibodies against pSMAD2 (**B, E, H,** arrowed) and CTGF (**N**, asterisk), show positive staining, co-localizing with the mineralization foci. In the whisker sections of the mouse model, no CTGF staining could be observed **(K)**. Staining of wild-type whisker **(C, L)**, eye **(F)**, and human skin tissues (**I** and **O**) did not show any staining. (n = 5 each). Scale bar = 100 μm.

qPCR expression analysis of the three ligands of the TGFβ family - TGFβ-1, TGFβ-2, TGFβ-3 - demonstrated increased expression of only TGFβ-2, while the others were within normal limits (Figure 6). After binding to a type II receptor dimer, TGFβ-2 is mediated by pSMAD2/3 and CTGF. To further confirm increased activity of TGFβ-2, qPCR of the critical downstream SMAD2, SMAD3 and CTGF gene in human PXE fibroblasts, and immunohistochemistry of critical mediators on murine and PXE tissues were performed. These experiments confirmed increased expression of the TGFβ-2 downstream signalling mediator CTGF (Figure 6). The expression of SMAD2 and SMAD3 was not significantly different compared to controls (Figure 6). Positive staining, co-localizing with mineralization, of pSMAD2 and CTGF was demonstrated in the PXE middermis (Figure 7, G-I and M-O respectively), and only pSMAD2 but not CTGF in the vibrissae and eye of the PXE KO mouse, compared to absent staining in controls (Figure 7, A-F and J-L respectively).

### Increased apoptosis in PXE tissues is only partially mediated by RUNX2

**Figure 8:**
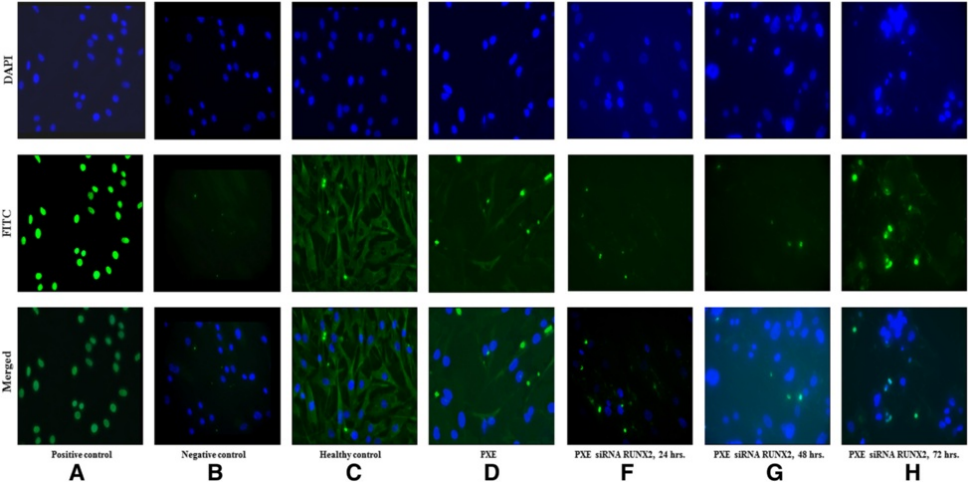
**Analysis of apoptosis by TUNEL staining in human fibroblasts (n = 6) compared to control cells (n = 5) (×20).** Counter staining was performed using DAPI nuclear staining and pictures were taken using DAPI (upper row), FITC (fluorescein isothiocyanate; middle row) and Merged (lower row) filters. Results are shown for a positive and negative control **(A, B)**, healthy control fibroblasts **(C)**, PXE fibroblasts **(D)** and PXE fibroblasts transfected with RUNX2 siRNA respectively 24 **(E)**, 48 **(F)** and 72 hours **(G)** following transfection. PXE fibroblasts show smaller nuclei on the DAPI stain and increased TUNEL staining in the cytoplasm of fibroblasts compared to controls, reflecting a three-fold increase of apoptosis (2,08% vs 0.69% respectively, p < 0.05). Twenty-four and 48 hours post-transfection with RUNX2 siRNA, a decrease of apoptosis can be seen, whereas after 72 hours no difference can be observed compared to untreated PXE fibroblasts. Scale bar = 20 μm.

**Figure 9:**
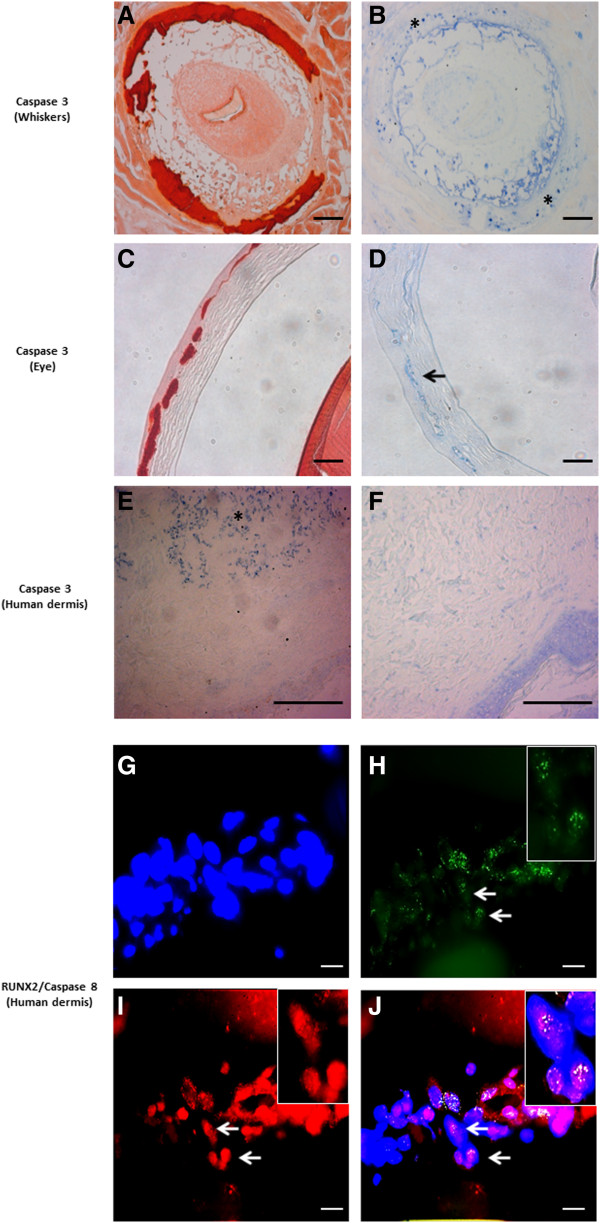
**Immunohistochemical staining results for Caspase 3 and Caspase 8.** Upper panel: Staining is performed on whisker and eye sections from the Abcc6 knockout mouse (**A-D,** ×10) and human skin (**E, F,** ×20). Alizarin Red stains was used to localize mineralization **(A, C)**. Positive staining for Caspase 3 is obtained on murine whiskers and eye (**B**, asterisk; **D**, arrow), co-localizing with ectopic mineralization. In human PXE dermis, Caspase 3 stains positive in the middermis (**E,** asterisk), while no staining is noted in controls **(F)**. Scale bar = 100 μm. Lower panel: Fluorescent immunohistochemistry for Caspase 8 and RUNX2 confirms co-localisation in PXE skin (**J,** white arrow). DAPI nuclear staining **(G)**. Merged RUNX2-DAPI image ((**H,** green fluorescence indicates RUNX2 staining, arrow) and merged Caspase 8-DAPI image (**I,** red fluorescence indicates Caspase-8 staining, arrow). (n = 5 each). Scale bar = 20 μm.

Apoptosis is considered an important mechanism in soft tissue mineralization. We evaluated the presence of apoptosis in PXE fibroblast cultures using a TUNEL assay, light and fluorescent IHC. DAPI staining revealed the nuclear diameter to be smaller in PXE fibroblasts compared to controls (Figure 8). After 72 hrs., TUNEL staining showed 3× more apoptosis compared to controls (2.08% and 0.69% respectively, p < 0.05) (Figure 8; Additional file 2: Figure S2). This staining did not always co-localize with the nuclear DAPI labelling, but was present in the cytoplasm of the cells (Figure 8; Additional file 3: Figure S3). Caspase 3 and Caspase 8 - both critical executioners of apoptosis -, were used as biomarkers for immunohistochemical evaluation of apoptosis [40]. IHC labelling of caspase 3 on murine whiskers and eye showed positive stains co-localizing with mineralization foci (Figure 9, A-D).

**Figure 10:**
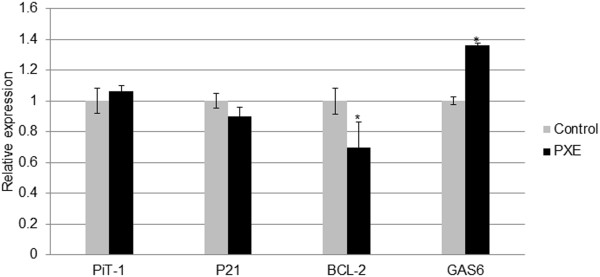
**qPCR result for mediators of apoptosis reported in vascular soft tissue mineralization (PiT-1, P21, BCL-2 and GAS6).** Relative expression shows a significant downregulation of BCL-2 and upregulation of GAS6 in human PXE fibroblasts compared to control cells. (n = 8 and 5 for patients and controls respectively). *p < 0.05.

**Figure 11:**
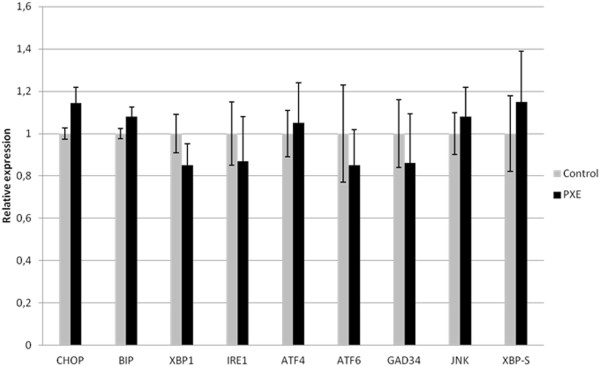
**qPCR results for genes involved in UPR dependent ER-stress and apoptosis including CHOP, BIP, XBP1, IRE1, ATF4, ATF6, GAD34, JNK, XBP-S.** Relative expression is shown in human PXE fibroblasts and control cells. No significant change in expression of any gene is observed. (n = 8 and 5 for patients and controls respectively). *p < 0.05.

Several signal transduction components have been attributed pro-apoptotic properties. RUNX2, P21, GAS6/BCL-2 and PiT-1 have all been involved in apoptosis-induced vascular calcification. Besides the upregulation of RUNX2 and GAS6, and downregulation of BCL-2, none of other mediators were found to show a significantly different expression pattern in PXE fibroblasts compared to controls (Figure 10). As also the Unfolded Protein Response (UPR), a cellular stress response originating from the ER, was shown to initiate apoptosis in vascular mineralization by inducing caspases, we evaluated several ER stress markers (CHOP, BIP, XBP1, IRE1, ATF4, ATF6, GAD34, JNK, XBP-S) in PXE fibroblasts but found none to be significantly different compared to healthy controls (Figure 11).

As RUNX2 was differentially expressed in PXE and to confirm its presence in the foci of apoptosis, we performed fluorescent IHC co-labelling with Caspase 8, confirming co-localisation of both antibodies (Figure 9, G-J). To further evaluate the contribution of RUNX2 to apoptosis, a siRNA experiment was performed to knock-down RUNX2 expression. Expression levels were downregulated by 67% after 24 hrs., diminishing to 51% and 31% after 48 and 72 hrs. respectively compared to cells treated with siRNA and untreated cells (Additional file 4: Figure S4). As TUNEL staining for apoptosis in PXE fibroblasts tends to have the highest yield after 72 hours, we mainly focused at the 48 h. time frame where there was still more than 50% downregulation of RUNX2. TUNEL staining on siRNA transfected fibroblasts showed a visual decrease of apoptosis after 24 and 48 hrs., which could not be seen anymore after 72 hrs. (Figure 8). Quantification of apoptosis in the transfected fibroblasts showed a reduction of 13 to 20% in the first 24 to 48 hrs., but with variable results between cell cultures (Additional file 5: Figure S5).

### pERK1/2 activation in PXE may be due to BMP2 effect

**Figure 12:**
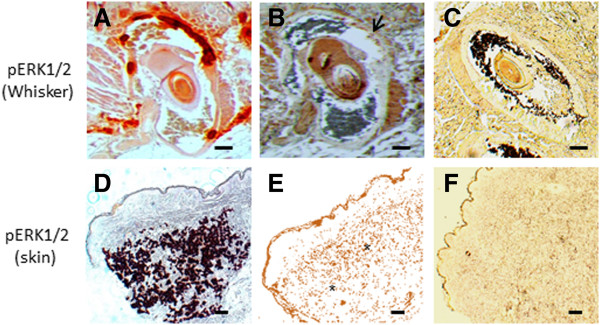
**Immunohistochemical staining results for pERK1/2 (×10).** Staining is performed on whisker from the Abcc6 knockout mouse **(A-C)** and PXE skin tissues **(D-F)**. Alizarin Red stains was used to localize mineralization **(A and D)**. Positive staining for pERK1/2 is obtained on murine whisker (**B,** arrow) and PXE skin (**E,** asterisk), co-localizing with mineralization. The pERK1/2 staining being broader than the mineralization staining could represent the active mineralization process where pERK1/2 expression precedes calcification. No staining is noted in control whisker **(C)** and control skin tissue **(F)**. (n = 5 each). Scale bar = 100 μm.

RUNX2-dependent transcription can also be regulated through the MEK/ERK pathway, either via BMP2 and/or PiT-1. We observed upregulation of pERK1/2, co-localizing with the calcified regions of both human PXE skin (middermis) and murine whiskers (Figure 12). PIT-1 expression was within normal limits (Figure 10).

## Discussion

Soft tissue mineralization is a complex process resulting from perturbation of a delicate interplay of developmental cues, protein signalling, transcription factors and their regulators. It is involved in both orphan and common disorders, leading to significant morbidity and mortality. Though still incompletely understood, important progress has been made in unravelling the signal transduction pathways leading to ectopic calcification. For this, vascular calcification has often been used as a model, in which the knowledge of cellular signalling resulted in improved understanding of the disease and novel therapeutic approaches [41,42]. Among orphan diseases, pseudoxanthoma elasticum is often considered a paradigm for ectopic mineralization disorders [43]. The pathophysiology of elastic fibre mineralization in PXE has so far remained unclear, though several mechanisms have been suggested to be involved, including chronic oxidative stress, an unidentified serum factor and deficiency of circulatory and local mineralization inhibitors [5,44]. Thus far, the data on cellular events in PXE are limited, restricted to specific tissues such as the heart and performed only in animal models [45]. It is unclear if and to what extent cellular mechanisms involved in vascular calcification are relevant in a multisystem mineralization disease such as PXE and more specifically in PXE patients. As it was recently demonstrated that these patients present a rather specific vasculopathy, differing from age-related atherosclerosis, it would seem presumptuous to automatically extrapolate the knowledge of calcified vasculopathies to PXE [16,46]. The pathobiological mechanisms in vascular calcification can be divided into two broad categories: induction of osteogenesis and loss of inhibitors of mineralization. Previous studies have already shown that several local and systemic calcification inhibitors, such as MGP and Fetuin-A, are functioning inadequately in PXE [5,15]. For the induction of an osteochondrogenic phenotype, the contribution of TGFβ signalling, BMPs-SMADs-RUNX2 signalling, Wnt-MSX2 signalling, apoptosis, oxidative stress and ER stress are well appreciated in calcified vasculopathies [3-9]. In this study, we wanted to assess which of these signalling pathways, if any, are perturbed in the PXE murine model and in PXE patients.

The TGFβ superfamily consists of a large number of members (TGFβs, activins, inhibins, nodals, anti-mullerian hormone and BMPs) involved in various biological processes such as cell proliferation, differentiation, migration, adhesion, apoptosis and ECM production [47,48]. In ectopic mineralization, particularly BMPs and TGFβs have been attributed a prominent role [49]. BMP2 and BMP4 are important propagators of ectopic mineralization in vessels through a concerted action with SMADs, and downstream key osteogenic transcription factors including RUNX2 (or Cbfa1), MSX2 and osterix (OSX) [40,50,51]. They can induce osteoblast differentiation in a variety of vascular cells [49]. BMP7 has been shown to inhibit vascular calcification [52]. Although Mungrue et al. described an upregulation of BMP4 in cardiac tissue of the Abcc6−/−mouse [37], we could not confirm involvement of BMP4 in PXE, though a tissue-specific effect cannot be fully excluded [53]. Comparably, BMP7 activity was also within normal limits in PXE, but BMP2 was considerably overexpressed in the whiskers and Bruch’s membrane of Abcc6 KO mice as well as in the mid-dermis and fibroblasts of PXE patients, co-localizing with the elastic fibre mineralization. BMP2 activity is regulated by MGP, which in its active, carboxylated form can prevent BMP2 to interact with its receptor [54]. The excess of uncarboxylated MGP in PXE may thus contribute to the upregulation of BMP2, though it is not excluded that other mechanisms, such as epigenetic changes, are also involved [20].

Though BMP2 is a crucial mediator of vascular calcification, its downstream affects are achieved through the upregulation of RUNX2, MSX2 and OSX [49-51]. Increased immunostaining of pSmad1, pSmad4, PSmad5, pSmad8 and pSmad1-5-8 and RUNX2 in PXE and Abcc6−/−murine tissues confirms that in PXE a coordinated activation of the BMP2- SMADs-RUNX2 signalling pathway occurs. RUNX2 is considered the master regulator of osteogenesis, although the gene is not osteoblast-specific [53]. Its upregulation has been observed in calcified vasculopathies, together with MSX2 and OSX, confirming its role in soft tissue mineralization, where it induces VSMC to acquire an osteogenic phenotype [20,54-56]. This osteogenic differentiation is obtained through induction of ALPL activity and expression of bone matrix protein genes OC, Col 1, and BSP [54,57]. Similar changes could be seen in PXE fibroblasts, with increased ALPL expression on qPCR and increased ALPL activity in human cultured fibroblasts. ALPL promotor activity may also be stimulated in a RUNX2-independent manner by DLX5 (Distal-less homeobox 5), but little DLX5 activity could be seen in PXE cells excluding this regulatory mechanism [58]. The mRNA expression of ALPL being considered a reliable method to determine BMP2-induced osteogenic differentiation of cells, this suggests that PXE fibroblasts adopt a gene expression profile similar to osteoblasts. It is also known that other target genes of RUNX2, such as OC or BSP, are abundantly present in PXE tissues [5,8]. Interestingly, RUNX2 has a dual effect on BSP. While normally inducing an overt BSP overexpression, RUNX2 has a tendency to decrease this BSP expression when upregulated itself. This might explain the observation of Contri et al. that BSP, though present, was significantly less abundant in PXE tissues compared to other pro-mineralizing proteins [21].

Besides induction by BMP2-SMADs, RUNX2-dependent transcription can also be regulated through the MEK/ERK (Extracellular Signal Regulated Kinases) pathway [59]. In vascular calcification, this is believed to pass through activation (phosphorylation) of ERK1 and ERK2, which can be achieved by both BMP2 and the phosphate transporter PiT-1. Though PXE tissues showed overexpression of pERK1/2, expression levels of PiT-1 were normal. Together with the normal phosphate levels in PXE patients, this excludes PiT-1 mediated ERK1/2 activation in PXE, but rather points towards an effect of BMP2. In epithelial cells, ERK1/2 activation has been shown to be precipitated by calcium influx [60]. It can thus not be excluded that the increased calcium in cultured PXE fibroblast, seen on Alizarin Red labelling, may serve as a positive feedback loop for ERK1/2 activity [61,62].

A second downstream mediator of BMP2 is MSX2, a transcription factor which promotes cardiovascular calcification by stimulating canonical Wnt signalling [31]. MSX2 induces nuclear stabilization of β-catenin resulting in a positive feedback of MSX2 expression and in the activation of TCF-1/LEF-1 transcription [31]. The upregulation of MSX2 and TCF-1/LEF-1 in PXE cells demonstrates involvement of MSX2-Wnt signalling in PXE. The diminished expression of DLX5, an important negative regulator of MSX2-Wnt signalling, may also contribute to MSX2 overexpression in PXE. Suppression of DLX5 is well-known in several embryological mechanisms. Though the precise mechanisms are ill-defined, the role of transcription factors and epigenetic factors such as microRNAs has been documented and may be tissue-specific [63,64]. The clinical importance of Wnt signalling has been shown for many disorders, where Wnt effectors can serve as susceptibility genes or modifiers [65]. The involvement of canonical Wnt signalling in PXE should thus encourage us to study Wnt-related pathways further.

The role and regulation of the third mediator of BMP2, OSX, is incompletely understood. OSX is a member of the Sp1 transcription factor family and plays an essential role in bone formation and osteoblastogenesis [66]. In soft tissue mineralization, OSX has been attributed an important role in the transdifferentiation of smooth muscle cells into osteoblasts. Initially thought to function downstream of RUNX2, OSX expression in osteoblasts was shown to be regulated by RUNX2-dependent and -independent mechanisms which are not necessarily simultaneously active [67]. Several factors such as OC, PTH, SP1 and DLX5 can influence OSX activity [68]. In PXE fibroblasts, OSX is not upregulated. The lack of OSX overexpression might explain why PXE fibroblasts, though they show an osteoblast gene expression profile, do not transform morphologically into osteoblasts.

The different TGFβ ligands (TGFβ-1, TGFβ-2, TGFβ-3) form a second group of TGFβ superfamily members which are implicated in vascular calcification [48]. TGFβ signalling in the vasculature is predominantly dependent on the activation of any of two type 1 receptors, Alk5 (TGFBR1) and Alk1 (ACVRL1), and signal transduction through activation of SMADs [69,70]. Among TGFβ ligands, TGFβ-1 is most frequently involved and promotes aortic smooth muscle cell calcification in culture, increases nodule formation in calcifying vascular cells in vitro, and is present in calcified aortic cusps [71-73]. Though TGFβ has already been suggested to be involved in PXE by Jiang et al., who reported that ABCC6 promoter activity can be modulated by several cytokines including TGFβ [74], it was somewhat unexpected to find only overexpression of TGFβ-2 in PXE fibroblasts, while expression of the other ligands (TGFβ-1 and TGFβ-3) remained the same as in controls. TGFβ-2 is expressed in the VSMCs of calcified arteriosclerotic arteries, induces chondrogenic differentiation of mesenchymal stem cells and induces calcifying activity in the human trabecular meshwork cells [75], but nearly always together with and less than TGFβ-1. Solitary TGFβ-2 overexpression seems to be unique in soft tissue mineralization diseases. Interestingly, isolated TGFβ-2 overexpression was found in models of choroidal neovascularisation, a complication often seen in PXE patients [76]. Further, the observed increased level of MMP2 and MMP9 in PXE serum may also reflect TGFβ-2 activity, as it has a pivotal role in activating both metalloproteases [77-79]. Involvement of TGFβ-2 in PXE was further confirmed by increased expression of its downstream effectors pSMAD2, pSMAD4 and CTGF (Connective Tissue Growth factor) [80,81]. CTGF is considered a mediator of TGFβ signalling in fibroblasts. In adult skin, CTGF is normally not expressed, unless induced for example during wound healing. The absence of CTGF labelling in the murine whiskers and eye may reflect a physio-immunological difference in between human and mice, rather than an actual difference in murine and human PXE pathogenesis.

Besides BMP2/TGFβ and Wnt signalling, also oxidative stress, endoplasmic reticulum stress and apoptosis can play a role in soft tissue calcification. PXE fibroblasts are well known to suffer mild chronic oxidative stress [82]. For all signalling pathways which are perturbed in PXE, a potential influence of reactive oxygen species (ROS) has been documented. There is some indication that BMP signalling may be mediated through ROS function and signalling [83]. ROS are able to induce both BMP2-RUNX2 signalling and MSX2-Wnt signalling, thus causing soft tissue calcification [84-86]. ROS-induced calcification was found to be retracted in RUNX2 knock-down cells, suggesting it to be RUNX2 dependent [87]. However, ROS has also been documented to influence those mediators which were normal in PXE, such as BMP4 [88]. This duality makes it less convincing that the contribution of ROS is a truly significant in PXE, though this remains difficult to assess.

The mechanisms of ER stress in vascular calcification include activation of the unfolded protein response (UPR), through unfolded protein sensors such as IRE1. UPR aims to restore normal ER function or, if not possible, aims towards apoptosis [89]. It involves a complex cascade of chaperone proteins (GRP78 and GRP94), activation of caspases, and induction of RUNX2 and OC via transcription factors ATF4 and XBP1s [90-93]. The presence of abundant and extremely dilated cisternae of the ER has been reported in PXE fibroblasts, as well as slightly modified GRP78 proteins [94-96]. Though this was suggestive for an involvement of ER stress, no significant change in expression level of ER stress markers previously implicated in vascular calcification (CHOP, IRE1, XBP1, ATF4 and ATF6) was noted in PXE fibroblast [97]. Based on this, we would conclude that ER stress does not play a major role in the PXE pathogenesis.

It is well known from calcified vasculopathies that an increased rate of apoptosis and apoptotic bodies function as a nidus for calcification [28]. Recent data have suggested that BMP2 and RUNX2 are pro-apoptotic factors [98,99]. The involved mechanisms depend on the cell-type. In soft tissue cells, they include downregulation of BCL-2, accumulation of cell-cycle arrest marker P21 and direct activation of Caspase 3, 8 and 9 [53,98,100]. In addition, increased levels of P and Ca^2+^, and oxidative stress also have a major role in apoptosis mediated soft tissue calcification [86,101]. On TUNEL staining for in situ cell death, PXE fibroblasts reveal a marked increase of apoptosis compared to controls. Apoptosis has never been reported in PXE, though the relation between Abcc6 deficiency and cell death has been reported in Abcc6 deficient mice with increased cardiac apoptosis and upregulation of BMP responsive transcription factors pSmad1/5/8 in the heart [37]. Of the three major mechanisms, PXE fibroblasts were noted to show a decreased BCL-2 expression and activation of Caspase 3 and Caspase 8. However the activity of P21 was not different in PXE fibroblasts compared to controls. Similar pro-apoptotic effects downstream of BMP2 were noted in pulmonary vascular smooth muscle cells [102,103]. There, BMP2 had the ability to increase caspase activity either directly or via RUNX2 [104,105]. The observation in PXE tissues that co-localization of Caspase 3 in mineralized area, and co-localization of RUNX2 with Caspase 8, suggested an important role for RUNX2 in PXE-related apoptosis. siRNA mediated silencing of RUNX2 demonstrated a decrease of apoptosis after 24 and 48 hours, though results were variable and apoptosis was still present despite RUNX2 downregulation. Though we did not achieve a complete siRNA-induced knockdown of RUNX2, this is in favour of a dual BMP2 and BMP2-RUNX2 effect as in other soft tissue cells. Whether other pro-apoptotic mechanisms are simultaneously at work in PXE cells remains to be determined.

Of interest is the observation that TUNEL staining in PXE fibroblasts is primarily present in the cytoplasm instead of the nucleus. This might be due to rapid degradation of the nuclear membrane due to cell death, though it has also been thought to reflect mitochondrial DNA damage [106,107]. As the positive control cells showed nuclear TUNEL staining, we consider it unlikely that the labelling in PXE fibroblasts is an artifact. This may further corroborate the involvement of mitochondria in the PXE pathophysiology, which was already suggested by previously reported ultra-structural and functional abnormalities of the mitochondria in PXE as well as the recent report of the presence of the ABCC6 transporter in the mitochondria-associated membranes [108,109]. However, further study is needed to assess to which extent mitochondrial dysfunction is implicated in the PXE pathogenesis.

## Conclusions

**Figure 13:**
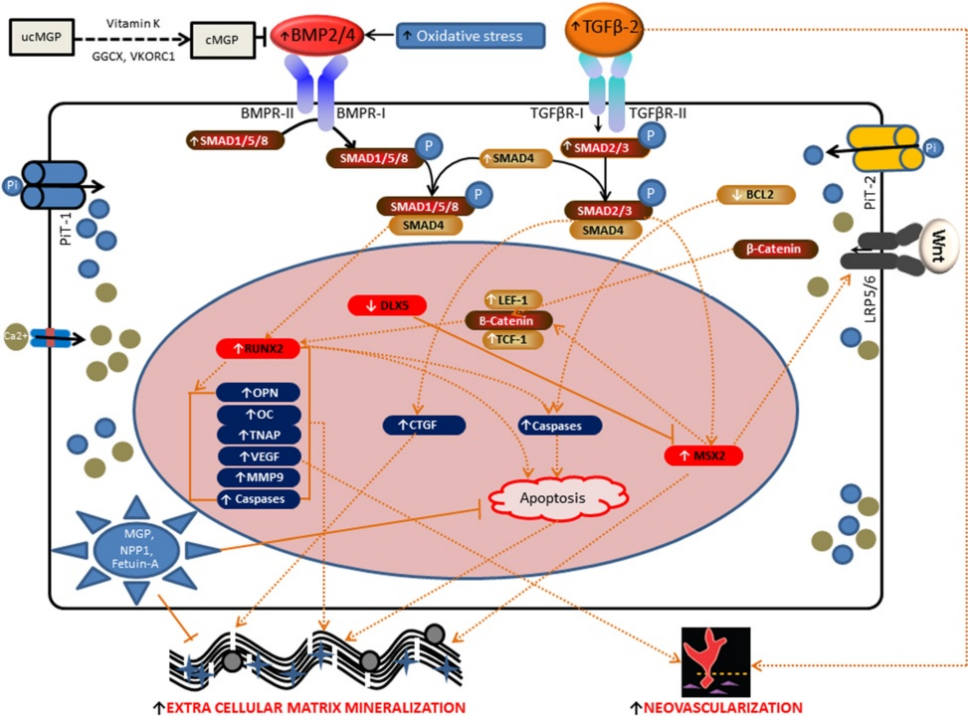
**Preliminary cell model of ECM calcification in PXE.** PXE fibroblasts reveal perturbation of a specific set of signalling pathways including BMP2-SMADs-RUNX2, TGFβ-2-SMADs-CTGF, MSX2-Wnt and pERK signalling. Apoptosis seems to be involved in PXE mineralization through RUNX2 and BCL-2. Besides the ECM mineralization, some of the perturbed signalling molecules, such as RUNX2 and TGFβ-2 can be related to neovascularisation, a common process in the complicated PXE retinopathy.

Our study shows that we cannot simply extrapolate knowledge on cell signalling in vascular soft tissue calcification to a multisystem ectopic mineralisation disease such as PXE. Contrary, we demonstrate a specific set of perturbed signalling pathways in PXE patients and the knock-out mouse model. Based on our findings and literature reports, we propose a preliminary cell model of ECM calcification in PXE (Figure 13). In the intricating web of calcification inhibitor and propagator signalling, this presents a starting point to further unravel the complex cellular signalling in PXE. Besides their relevance for elastic fibre mineralization, the pathways that we found disturbed may have a relevance for other features of the PXE phenotype, such as neovascularisation. PXE patients often suffer subretinal neovascularisation and haemorrhaging as a complication of the PXE retinopathy [110,111]. VEGF-A, a potent mediator of angiogenesis, is assumed to play a role in this neo-angiogenesis, as treatment of PXE patients with anti-VEGF injections has been very successful [112]. However, the mechanism behind VEGF-A involvement has not yet been uncovered. Runx2 has been described to induce overexpression of Vegf-A and the upregulation of Runx2 in Bruch’s membrane of the PXE KO mouse model, may contribute to the presence of VEGF-A in the eye [52]. Also, the TGFβ-2 predominance in experimental models of choroidal neovascularization may provide further insights in the PXE retinopathy [71]. These results may also have implications for future therapies for this as yet intractable disease. Curative treatments targeting just one of the biochemical abnormalities in PXE, being it e.g. vitamin K deficiency or oxidative stress, might not be sufficient because of their synergistic effect [69,70,113]. Conversely, a more central component such as RUNX2 might provide a powerful target to influence these different mechanisms, as well as the BMP2 receptor. Indeed, several small-molecule inhibitors of BMP receptors have been developed and mouse models of ectopic mineralization diseases such as Fibrodysplasia Ossificans Progressiva, where BMP pathways are deregulated, have already been successfully treated with these compounds [114,115].

## Competing interests

The authors’ declare that they have no competing interests.

## Authors’ contribution

MJH and OMV contributed to the experimental design, data collection and interpretation. PJC contributed to the data collection and interpretation. OLS provided the mouse specimens and contributed to the data interpretation. ADP was involved in study design and data interpretation. All authors were involved in writing the paper and had final approval of the submitted and published versions. MJH, ADP and OMV accept responsibility for the integrity of the data analysis.

## Supplementary Material

Additional file 1 List of primers used in qPCR experiments. Click here for file

Additional file 2 **Rate of apoptosis (%) in PXE fibroblasts compared to controls.** PXE fibroblasts showed 3× more apoptosis compared to controls. (n = 8 and 5 for patients and controls respectively). Click here for file

Additional file 3 **Magnification of TUNEL staining in native PXE fibroblasts (×40).** Positive labelling in the cytoplasm of the cells is arrowed. Scale bar = 50 μm. Click here for file

Additional file 4 **Relative expression of RUNX2 in PXE fibroblasts transfected with anti-RUNX2 siRNA after 24, 48 and 72 hours.** siRNA silencing causes a downregulation of RUNX2 expression of 65% at 24 hours, 54% at 48 hours and 28% at 72 hours. (n = 6 each). Click here for file

Additional file 5 **Quantification of the effect of RUNX2 knockdown on apoptosis in PXE fibroblasts (n = 2 cultures).** Results are shown after 24, 48 and 72 hours respectively and demonstrate a 13 to 20% reduction of apoptosis, though results differ between different cell lines. After 72 hours, no reducing effect of siRNA silencing of RUNX2 on apoptosis can be seen anymore. Click here for file
