# Supplementary figures and images for: Perturbation of specific pro-mineralizing signalling pathways in human and murine pseudoxanthoma elasticum
Source: Orphanet J Rare Dis. 2014 Apr 29;9:66. doi: 10.1186/1750-1172-9-66 (PMC4022264; doi:10.1186/1750-1172-9-66)

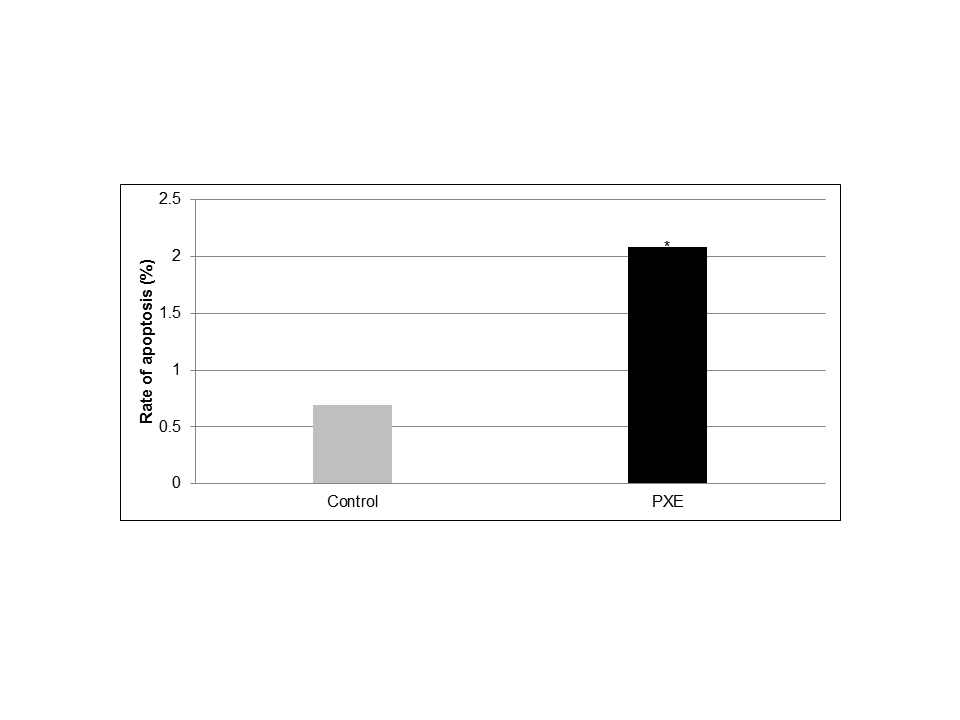

Supplement: Additional file 1 — List of primers used in qPCR experiments. [file 1750-1172-9-66-S1.tiff]

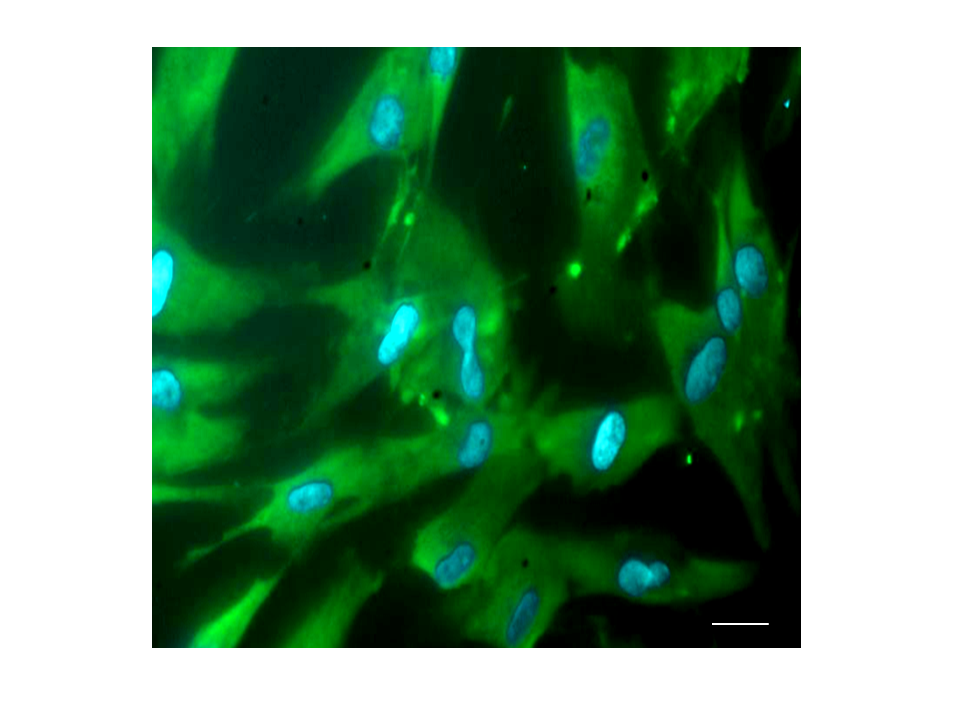

Supplement: Additional file 2 — Rate of apoptosis (%) in PXE fibroblasts compared to controls. PXE fibroblasts showed 3× more apoptosis compared to controls. (n = 8 and 5 for patients and controls respectively). [file 1750-1172-9-66-S2.tiff]

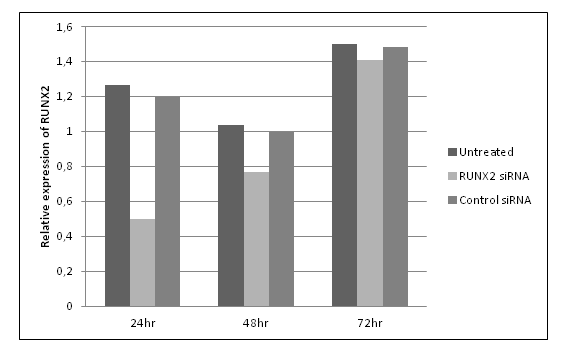

Supplement: Additional file 3 — Magnification of TUNEL staining in native PXE fibroblasts (×40). Positive labelling in the cytoplasm of the cells is arrowed. Scale bar = 50 μm. [file 1750-1172-9-66-S3.tiff]

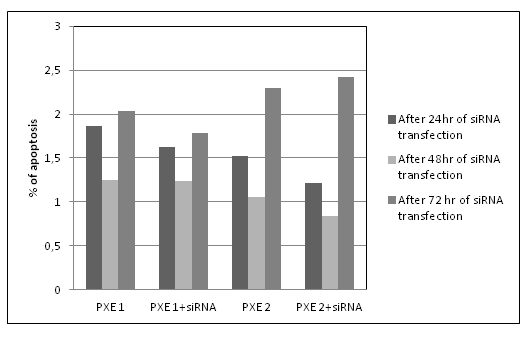

Supplement: Additional file 4 — Relative expression of RUNX2 in PXE fibroblasts transfected with anti-RUNX2 siRNA after 24, 48 and 72 hours. siRNA silencing causes a downregulation of RUNX2 expression of 65% at 24 hours, 54% at 48 hours and 28% at 72 hours. (n = 6 each). [file 1750-1172-9-66-S4.tiff]
