# Supplementary material for: Perturbation of specific pro-mineralizing signalling pathways in human and murine pseudoxanthoma elasticum
Source: Orphanet J Rare Dis. 2014 Apr 29;9:66. doi: 10.1186/1750-1172-9-66 (PMC4022264; doi:10.1186/1750-1172-9-66)
Supplement: Additional file 5 — Quantification of the effect of RUNX2 knockdown on apoptosis in PXE fibroblasts (n = 2 cultures). Results are shown after 24, 48 and 72 hours respectively and demonstrate a 13 to 20% reduction of apoptosis, though results differ between different cell lines. After 72 hours, no reducing effect of siRNA silencing of RUNX2 on apoptosis can be seen anymore. [file 1750-1172-9-66-S5.doc]

List of primers used in qPCR experiments

| Gene | Forward primer | Reverse primer |
| --- | --- | --- |
| RUNX2 | GTAGCAAGGTTCAACGATCT | GTGAAGACGGTTATGGTCAA |
| LEF-1 | TGGCATCCCTCATCCAGCTATTGT | TTTGGCTCCTGCTCCTTTCTCTGT |
| TCF-1 | TGACCTCTCTGGCTTCTACT | TTGATGGTTGGCTTCTTGGC |
| ALPL | GACCCTTGACCCCCACAAT | GCTCGTACTGCATGTCCCCT |
| MSX2 | TTCTCAAGGCTGTTGGTAAC | CCCAAATCACCTTTGCAATC |
| BMP4 | ACCATCAGCATTCGGTTACCAGGA | TGACCACCTCAACTCAACCAACCA |
| TGFβ-1 | CGGGTTATGCTGGTTGTA | GGACACCAACTATTGCTTCA |
| TGFβ-2 | TGCCCTACTTGTGCTTTGTGTTTCT | AGACCACTGAACTCGAACCCATCT |
| TGFβ-3 | GGAATTCTGCTCGGAATAGG | TGCCAAAGAAATCCATAAATTCG |
| CTGF | CCACCCGAGTTACCAATGAC | GACAGGCTTGGCGATTTTAG |
| SMAD1 | CAGAGCCACCATGAACTAAA | ACAGGGCTTTCTACTCTCTT |
| SMAD2 | GGCAATTGAAAACTGCGAAT | GAGGTGGCGTTTCTGGAATA |
| SMAD3 | CCCCAGAGCAATATTCCAGA | GGCTCGCAGTAGGTAACTGG |
| SMAD4 | TGTGCCATAGACAAGGTGGA | AGCCTCCCATCCAATGTTCT |
| SMAD5 | AAAGCCGTTGGATATTTGTGA | GAAGGCTGTGTTGTGGATTG |
| SMAD8 | GTGCCAAGACACAGTGAATA | TGCTGGAAAGAGTCAGGATA |
| PiT-1 | CGTGCATTCATCCTCCATAA | CAACTGTGCAGGCATAGAAA |
| P21/CDKN1 | AGACCAGCATGACAGATTTC | ACTGAGACTAAGGCAGAAGA |
| BCL-2 | GGTCATGTGTGTGGAGAGC | GATCCAGGTGTGCAGGTG |
| GAS6 | AACCATGGCATGTGGCAGACAATC | TCATGACAGCATCCCTGTTGACCT |
| CHOP | AAGGCACTGAGCGTATCATGT | TGAAGATACACTTCCTTCTTGAACA |
| XBP1 | TTACGAGAGAAAACTCATGGCC | GGGTCCAAGTTGTCCAGAATGC |
| IRE1 | TTTGGAAGTACCAGCACAGTG | TGCCATCATTAGGATCTGGGA |
| ATF4 | CAGACTACACTGCTTACGTT | ATAGGACTCTGGGCTCATAC |
| ATF6 | TCAGACAGTACCAACGCTTATGC | GTTGTACCACAGTAGGCTGAGA |
| GAD34 | TCCTCTGGCAATCCCCCATA | GGAACTGCTGGTTTTCAGCC |
| JNK | AGGAGAGAACCAAGAATGGA | AGTCGGATCTGTTGACATTG |
| XBP-S | AGACAGCGCTTGGGGATGGAT | AGTGTCCTCCCAAGAATGGTTTA |
